# Open Hip Abductor Repair Hitting the Sack-Abductor Tendon Repair Significantly Improves Sleep Quality

**DOI:** 10.3390/jcm10215164

**Published:** 2021-11-04

**Authors:** Alexander Zimmerer, Luis Navas, Dominic Pfeil, Matthias Hauschild

**Affiliations:** 1 ARCUS Sportklinik, Rastatterstr. 17-19, 75179 Pforzheim, Germany; navascontreras@sportklinik.de (L.N.); dominicpfeil@gmail.com (D.P.); hauschild@sportklinik.de (M.H.); 2 Department of Orthopaedics and Orthopaedic Surgery, University Medicine Greifswald, Ferdinand-Sauerbruch-Straße, 17475 Greifswald, Germany

**Keywords:** hip, abductor repair, gluteus medius, sleep quality, MCID

## Abstract

Purpose: To (1) describe the prevalence of abnormal sleep quality in patients with hip abductor tears (HAT), to (2) determine whether sleep quality improves after open HAT repair, and to (3) to report clinical short-term outcomes in patients undergoing open HAT repair. Methods: The data of 28 patients (29 hips) who underwant open HAT repair were prospectively analyzed at midterm follow-up. The Pittsburgh Sleep Quality Index (PSQI), modified Harris Hip Score (mHHS), the University of California, Los Angeles activity scale (UCLA), and Visual Analog Scale (VAS) for pain were determined via questionnaire. Paired t-tests were applied to compare preoperative and post-operative Patient-reported Outcome Measures (PROMs). Logistic regression was performed to determine the association between PSQI improvement achievement and demographic variables (laterality, sex, age, body-mass-index (BMI), and preoperative mHHS). The minimal clinically important difference (MCID) was calculated for the mHHS. Results: A total of 28 patients were included. Four patients (14.3%) suffered post-operative complications after open HAT repair. The predominance of patients was female (77.4%), with a mean age of 60 ± 13 years. The average follow-up was 30.35 ± 16.62 months. Preoperatively, 27 (96.4%) patients experienced poor sleep quality (PSQI > 5); at follow-up, 7 (25%) patients experienced poor sleep quality. Univariate logistical regression analysis demonstrated no significant association between preoperative demographic data and achieving postoperative PSQI < 5. The MCID of mHHS was calculated to be 12.5. Overall, 90% of patients achieved MCID for mHHS. Conclusion: Preoperative sleep quality was impaired in 96.4% of HAT patients (PSQI > 5). However, these patients showed an improvement in sleep disturbances after open HAT repair in the early postoperative period. Ninety percent of patients showed significant improvements in mHHS and achieved the corresponding MCID. Level of Evidence: Case series; Level IV.

## 1. Background

Hip abductor tear (HAT) is an increasingly diagnosed cause of refractory lateral hip pain and dysfunction in the aging population [1,2,3,4,5,6]. HAT is usually caused by direct trauma, iatrogenic injury during hip surgery (e.g., total hip arthroplasty (THA) using direct lateral approaches), which can lead to degenerative damage and/or tissue damage by metal ions in metal-on-metal THA [7,8,9,10]. Patients with HAT present with lateral hip pain, hypersensivity to palpation of the greater trochanter, debilitated hip abduction against resistance, and a positive Trendelenburg sign. These symptoms are excacerbated by walking long distances, walking up and down stairs, or sleeping on the affected side [11,12,13]. With conservative therapy failing, the option of open or endoscopic surgical treatment of HAT is considered. A systematic review compared open and endoscopic procedures and showed a statistically significant improvement in outcomes and reductions in pain for both methods [2]. Recent publications have further shown that approximately 80% of surgically treated patients achieved the minimal clinically important difference (MCID) for the modified Harris Hip Score (mHHS) after open or endoscopic HAT repair [13,14].

Nocturnal pain has been shown to affect sleep quality, whereby good sleep quality is associated with a variety of positive outcomes, such as better health, reduced daytime sleepiness, improved well-being, and better psychological performance. Thus, sleep disturbances are among the common symptoms associated with almost all psychosomatic, neurological, cardiovascular, respiratory, and metabolic disorders, as well as acute and chronic pathological conditions [15,16,17,18,19,20,21,22]. The impact of varying musculoskeletal pathologies on sleep quality has been thoroughly investigated [23,24,25,26]. A recent study has shown that sleep disorders are very commonly found in patients with femoroacetabular impingement syndrome (FAIS). These patients experienced an improvement improvement in sleep disturbances after arthroscopic hip surgery [23].

Further studies have investigated the effect on sleeping after THA and demonstrated that THA significantly improves sleep quality postoperatively [24,25,26]. Given those patients with hip joint pathologies, it is conceivable that patients with HAT suffer from sleep disturbances, one of the most common complaints of patients with symptomatic abductor pathology.

Although functional outcomes of HAT repair have been reported [2,13,14], the effect of HAT repair on sleep quality has not yet been studied. The aim of this study was to (1) describe the prevalence of abnormal sleep quality in patients with HAT, to (2) determine whether sleep quality improves after open HAT repair, and to (3) to report clinical short-term outcomes in patients undergoing open HAT repair. We hypothesized that sleep disturbances are a common problem in patients attending our institution with full-thickness HAT and that repair would lead to a significant improvement in sleep quality after surgery.

## 2. Methods

### 2.1. Patient Selection

This is a single-center cohort study. After institutional review board approval (Ethikkommission der Landesärztekammer Baden-Würtemmberg, Germany, F-2019-006), a consecutive series of patients undergoing open hip abductor repair by the senior author (M.H.) between March 2016 and March 2020 were reviewed. We identified patients via our institutional database and performed a retrospective analysis of prospectively collected data via questionnaire. Patients were asked for their consent to participate in the study and for their pre- and post-operative data to be prospectively recorded in a secure institutional repository.

**Figure 1:**
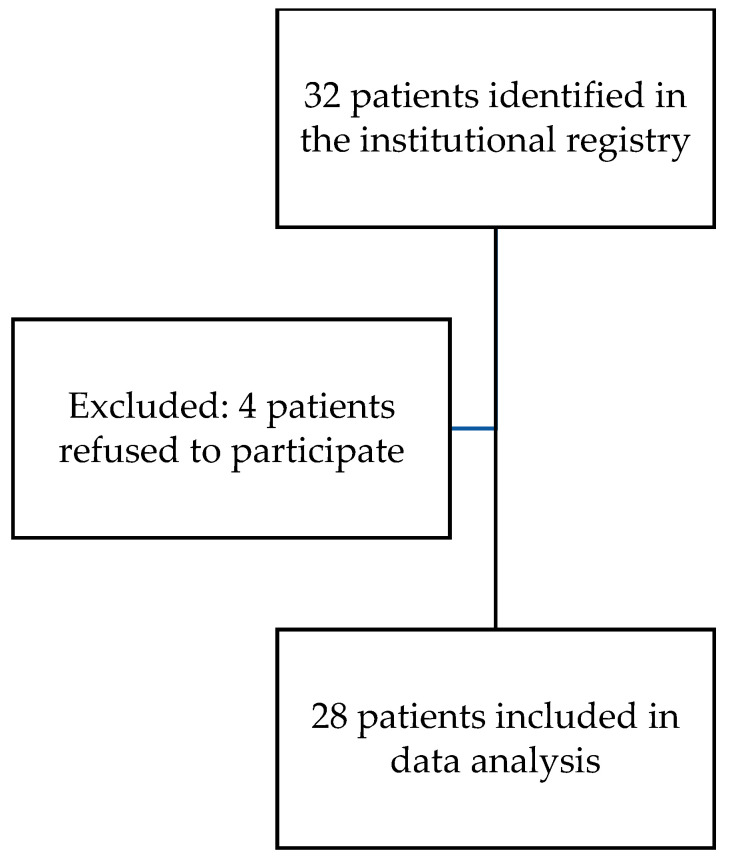
Patient inclusion/exclusion flowchart.

Indications for open HAT refixation included lateral hip pain, debilitated abduction on physical examination, magnetic resonance imaging (MRI) findings compatible with full-thickness gluteus medius and/or minimus tear, and failure of at least 6 months of non-operative therapy, including non-steroidal anti-inflammatory drugs (NSAIDs), platelet-rich plasma (PRP) infiltration, and physiotherapy. Exclusion criteria included a history of pediatric hip malformatios, prior surgery of ipsilateral HAT, partial-thickness gluteus medius and/or minimus tears, a follow-up period shorter than 12 months, or inability to consent to the study. Complication data were collected by reviewing the electronic medical records at our facility. The clinical examination was performed by two fellowship-trained orthopedists (M.H., A.Z.). Likewise, MR imaging was assessed by both examiners. The data were analyzed by calculating intraclass correlation coefficients (ICCs). We found excellent agreement between the two observers in classifying the tear types (ICC, 0.98). The patient enrollment flowchart is demonstrated in Figure 1.

### 2.2. Surgical Technique

The technique performed was recently described [27]. All operations were conducted by the senior author (M.H.) with the patient under general anesthesia in a lateral decubitus position. A standard lateral approach was utilized via a 6–8 cm longitudinal skin incision focalized over the greater trochanter and the iliotibial band (ITB). After entering the peritrochanteric space with an incision through the ITB, the trochanteric subgluteus bursa was removed, and once the tear was identified, a longitudinal splitting of gluteal tendons over the tear was performed. Then debridement and mobilization of tendons for sufficient distalisation to the tendon footprint at anterior and lateral trochanteric facet, debridement of sclerosis on the great trochanter, punching, and tapping the proximal anchor row was performed. The placement of two proximal 3.5 mm SwiveLock anchors (Arthrex, FL, USA) loaded with non-resorbable suture tapes was then performed. The suture tapes were then passed through the tendon in a fan-shaped manner. After passing, the suture tapes were crossed in a double-v shape and fixed with 4.75 mm SwiveLock anchors in the distal row under mild pre-tensioning of the gluteal tendons. The ITB was closed with 2-vicryl sutures. The subcutaneous tissue was also closed with 2-0 vicryl sutures, and the skin was closed with a running subcuticular 3-0 monocryl suture. The hip was gently adducted and abducted using a brace to ensure adequate tensioning of the repair.

### 2.3. Post-Operative Management

The postoperative recovery program was uniform for all patients. Patients were given a hip brace during the first 6 postoperative weeks to limit abduction and external rotation. Partial weight-bearing was allowed at 20 kg. Patients were allowed full weight-bearing during the following 6 weeks and began hip-strengthening exercises while the brace was removed. After these 12 weeks, patients were allowed to walk unassisted and return to activities they tolerated in a pain-adapted manner. Deep venous thrombosis prophylaxis was recommended until full weight-bearing was reached.

### 2.4. Sleep Quality Assessment

To assess sleep disturbances in patients who underwent a surgical HAT repair, the Pittsburgh Sleep Quality Index (PSQI) was applied to all patients participating in this study [28]. The PSQI is a validated and widely used questionnaire of 9 question divided into seven scorable subcomponents that assess sleep quality and sleep patterns. It defines sleep quality by measuring the following seven components: perceived sleep quality, sleep latency, sleep duration, sleep efficiency, sleep disturbances, use of sleep aids, and daytime sleepiness in the past month. The PSQI total score (sum of seven component scores) ranges from 0 to 21, with an increasing score ≥ 5 indicating a deterioration in sleep quality, while <5 indicates normal sleep quality [28]. PSQI was collected preoperatively and at the last follow-up.

### 2.5. Patient-Reported Clinical Outcomes

The mHHS [29], and the University of California, Los Angeles activity scale (UCLA) [30] surveys were assessed preoperatively and at the last follow-up. Patients described a visual analog scale (VAS) for pain at these time points. To quantify the clinical significance of meaningful outcome among patients who underwent open HAT repair, the MCID was estimated using a half standard deviation (distribution-based) method of change in mHHS pre- and postoperatively.

### 2.6. Statistical Analysis

Means and standard deviations were reported for continuous variables. According to the Shapiro–Wilk test, the study cohort was normally distributed (*p* = 0.257). Differences between pre-and postoperative data were examined with a paired *t*-test and Wilcoxon signed-rank test. McNemar’s test statistic was conducted to detect differences. A univariate logistic regression analysis was conducted to assess the association between PSQI improvement achievement and demographic variables (laterality, sex, age, body-mass-index (BMI), and preoperative mHHS). Statistical analyses were conducted using SPSS statistical software (IBM SPSS Statistics for Windows, version 26.0.0; IBM Corp, Armonk, NY, USA).

## 3. Results

A total of 28 patients (1 bilateral HAT repair) were enrolled in the study. The majority of patients were female (*n* = 24; 77.4%). The mean age was 59.79 ± 12.45 (29–85) years, and the mean BMI was 27.99 ± 4.45 (20.24–35.29) kg/m^2^ (Table 1). The mean follow-up was 30.35 ± 16.62 (12–57) months. Re-tear occurred in three hips during the follow-up, and one surgical site infection was observed in one patient, which required surgical intervention.

Preoperatively, 27 (96.4%) patients reported a PSQI score > 5 points, corresponding to poor sleep quality, and postoperative 6 patients (25%) described a PSQI score > 5 points (*p* < 0.0001).

Univariate logistical regression analysis demonstrated no significant association between preoperative demographic data and achieving postoperative PSQI < 5 (Table 2).

Analysis of pre- and post-operative outcomes showed statistically significant improvements in PSQI (13.7 ± 4.6 vs. 4.2 ± 3.8; *p* < 0.0001), mHHS (28.6 ± 13.7 vs. 71.6 ± 28.3; *p* < 0.0001), UCLA (3.9 ± 1.7 vs. 5.0 ± 1.5; *p* = 0.007), and VAS (8.9 ± 1.1 vs. 3.14 ± 2.6; *p* < 0.0001) scores (Table 3). The MCID threshold of mHHS was 12.5, and a total of 25 patients (90%) achieved MCID for the mHHS.

## 4. Discussion

Our study demonstrated that 96.4% of open HAT repair patients experienced abnormal sleep quality (PSQI > 5). However, these patients showed an improvement in sleep disturbances in the early postoperative period. Of the variables assessed here, no preoperative factors could be identified affecting the achievement of sleep improvement. The MCID for mHHS was calculated to be 12.5 points, with 90% of patients achieving it. 

The association between joint pathology, sleep disturbances, and postoperative outcomes is becoming increasingly apparent [12,23,24,25,31,32,33,34,35]. According to the generally accepted criterion for bad sleep quality (PSQI > 5 [28]), approximately 35% of the German population [36,37] and 40% of the world population [38] sleeps poorly. Austin et al. found that 89% of their patients had abnormal sleep quality preoperatively, and that 6 months after arthroscopic shoulder rotator cuff repair, only 38% of patients had abnormal sleep quality. In this study, the authors determined that their patient population was three to six times more likely than the general population to complain of sleep disturbances preoperatively [31]. Kunze et al. determined that 94.2% of their patients reported abnormal sleep quality preoperatively and that at 6 months after hip arthroscopy for FAIS, only 21.7% of patients described anomalous sleep quality [23]. Our study is in line with these findings and confirms that joint pathologies may impact sleep quality. In total, 96.4% of our patients reported abnormal sleep quality, approximately three times that of the average German population. Nevertheless, open surgical treatment of full-thickness HAT improved sleep quality in the short-term follow-up significantly. These findings are significant because there is a lack of evidence regarding the association between sleep quality and HAT. We did not identify any preoperative patient-specific factors that influenced improved sleep quality. Future studies are necessary to better define the etiology of these sleep disturbances in these patients. Multiple studies have shown that open hip abductor repair is linked with statistically significant improvements in functional and pain scores as well as in PROMs [14,39,40,41]. Davies and Davies described significant improvements in Lower-Extremity Activation Score and mHHS at an average follow-up of 71 months [39]. Uppstrom et al. defined MCID for mHHS (9.9) and International Hip Outcome Tool (iHOT-33) score (14.3) and demonstrated significant improvements on the mHHS and iHOT-33 scores at an average follow-up of 37.8 months [14]. These results are compatible with the postoperative improvements in the mHHS score demonstrated in our study. However, only Uppstrom et al. addressed the proportion of patients achieving MCID for their PROMs, which is slightly below our results (82.9% vs. 90%). We also found that the sports activity in our patients assessed with the UCLA activity scale slightly improved from 3.9 preoperative to 5 postoperative.

Recently, endoscopic HAT repair has been increasingly used since several studies have raised concerns about the increased complication rates with open techniques. Alpaugh et al. and Chandrasekaran et al. reported systematic reviews researching endoscopic versus open HAT repairs [2,12,32]. The authors of both reviews reported good to excellent outcomes in 75% of the patients for both endoscopic and open HAT repairs. Complication rates were 3% for endoscopic and 13% for open repairs, with re-rupture rates of 0% for endoscopic and 10% for open repairs, although heterogenicity of complication reports among the selected studies and selection bias are important limitations, considering that several endoscopic repair studies reported no re-rupture rates and may be biased in favor of smaller HAT compared to open repairs [2,12,32]. In our study, the complications rate was 14.3%, with a re-rupture rate of 10%, both of which are lower than rates reported for open repair in the previously mentioned systematic reviews.

## 5. Limitations

Our study is not free of limitations. First, our sample is a small case series, which may impede statistical significance. Second, this study lacks a control group to determine if the open HAT repair is the only or principal reason for improved sleep quality. Also, control groups of patients without a diagnosis of HAT and matched by age would help assess in perspective the prevalence of preoperative sleep disturbances of HAT patients. However, we believe that the high-resolution rate of poor sleep quality in the postoperative period is strong evidence that open HAT repair played an essential role in improving sleep.There was also a high proportion of female patients in our cohort, limiting the magnitude to male patients. However, these results are consistent with previous studies [1,11,42] and demonstrate the higher prevalence of this pathology in female patients. To date, the PSQI has not yet been fully implemented in orthopedic surgery, however, it is used in the majority of studies examining sleep quality [23,24,25,26]. Other measurement methods, such as actigraphy, can objectively measure sleep quality, but cannot determine other psychological or pharmacological aspects of sleep reconciliation. Similarly, other studies such as the Epworth Sleepiness Scale, which is a subjective measurement scale, have the problem of being a daytime sleep measurement method.The reason we have decided on a single scale is that we have been guided by other orthopaedic studies that have used the same scale for this measurement, and so far it is the most reliable scale for measuring sleep quality in orthopedic and arthroscopic surgery.

## 6. Conclusions

Preoperative sleep quality was impaired in 96.4% of HAT patients (PSQI > 5). However, these patients showed an improvement in sleep disturbances after open HAT repair in the early postoperative period. Ninety percent of patients showed significant improvements in mHHS and achieved the corresponding MCID.

## Figures and Tables

**Table 1:** Patient demographic data.

	Value
Total no. of patients	28 (29 Hips)
Laterality, *n*(%)	
Right	15 (52%)
Left	14 (48%)
Gender, *n* (%)	
Female	24 (85.7%)
Male	4 (14.3%)
Age, y	59.79 ± 12.45 (29–85)
Body mass index, kg/m^2^	27.99 ± 4.45 (20–35)

Values are shown as *n* (%) or mean ± SD (range).

**Table 2:** Logistic Regression Analysis of Preoperative Variables Associated with Achieving PSQI < 5.

.	*p*-Value Univariate	*Odds Ratio*	95%-CI
Laterality	0.758	1.023	0.823–1.103
Sex	0.368	1.123	0.825–1.123
Age	0.598	0.925	0.815–1.051
BMI	0.698	0.987	0.727–1.341
Preoperative mHHS	0.658	0.863	0.742–1.003

BMI, Body Mass Index; CI, confident interval; mHHS, modified Harris Hip Score; PSQI, Pittsburg Sleep Quality Index.

**Table 3:** Pre- and Post-operative Patient-Reported Outcomes.

Score	Preoperative	Postoperative	*p*-Value
PSQI *	13.7 ± 4.6 (4–21)	4.2 ± 3.8 (1–20)	<0.0001
mHHS ^§^	28.6 ± 13.7 (5.5–59.4)	71.6 ± 28.3 (8.8–95.7)	<0.0001
UCLA ^§^	3.9 ± 1.7 (1–9)	5.0 ± 1.5 (2–8)	0.007
VAS ^§^	8.9 ± 1.1 (7–10)	3.14 ± 2.6 (0–9)	<0.0001

Values are shown as *n* (%) or mean ± SD (range). *, statistics was performed by means of Wilcoxon signed-rank test. §, statistics was performed by means of t-test. mHHS, modified Harris Hip Score; PSQI, Pittsburg, Sleep Quality Index; UCLA, the University of California and Los Angeles activity scale; VAS, Visual Analog Scale for pain.

## Data Availability

The data presented in this study are available on request from the corresponding author.
